# 3-(6-Fluoro-4-oxo-4*H*-chromen-3-yl)-3,4-dihydro-2*H*-1,2,4-benzothia­diazine-1,1-dione

**DOI:** 10.1107/S1600536810038274

**Published:** 2010-10-02

**Authors:** Mariya al-Rashida, Saeed Ahmad Nagra, Islam Ullah Khan, George Kostakis, Ghulam Abbas

**Affiliations:** a Institute of Chemistry, University of the Punjab, Lahore, Pakistan; b Department of Chemistry, Government College University, Lahore, Pakistan; c Institute of Inorganic Chemistry, Karlsruhe Institute of Technology, D-76133 Karlsruhe, Germany

## Abstract

In the title compound, C_16_H_11_FN_2_O_4_S, the mean planes of the bicyclic chromone system and of the benzene ring of the benzothia­diazine derivative make a dihedral angle of 54.28 (5)°. An intra­molecular N—H⋯O hydrogen bond occurs. In the crystal, mol­ecules are linked into layers by N—H⋯O and C—H⋯O hydrogen bonds, generating an infinite two-dimensional network.

## Related literature

For background to the importance of the 1,2,4-benzothia­diazine-1,1-dioxide ring system in pharmaceutical and medicinal chemistry, see: Zhu *et al.* (2005 ▶); Kamal *et al.* (2007*a* ▶). For a survey on the anti­microbial activity of benzothia­diazine derivatives, see: Di Bella *et al.* (1983 ▶); Kamal *et al.* (2007*a* ▶,*b* ▶). The sulfonamide group is an active pharmacophore, see: Weisman & Brown (1964 ▶). For a related structure, see: Mariya-al-Rashida *et al.* (2009 ▶);
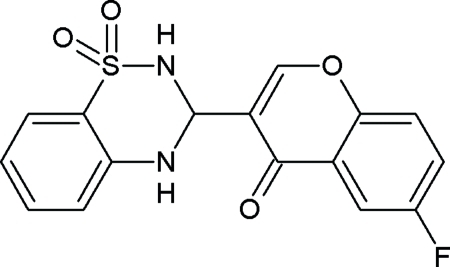


## Experimental

### 

#### Crystal data

C_16_H_11_FN_2_O_4_S
*M* *_r_* = 346.34
Orthorhombic, 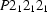

*a* = 7.0739 (3) Å
*b* = 8.2861 (4) Å
*c* = 25.0456 (12) Å
*V* = 1468.05 (12) Å^3^
*Z* = 4
Mo *K*α radiation
μ = 0.26 mm^−1^
*T* = 296 K
0.31 × 0.06 × 0.05 mm

#### Data collection

Bruker APEXII CCD area-detector diffractometer
9538 measured reflections
3453 independent reflections
1993 reflections with *I* > 2σ(*I*)
*R*_int_ = 0.056

#### Refinement

*R*[*F* ^2^ > 2σ(*F* ^2^)] = 0.052
*wR*(*F* ^2^) = 0.093
*S* = 0.97
3453 reflections
223 parameters
3 restraints
H atoms treated by a mixture of independent and constrained refinement
Δρ_max_ = 0.23 e Å^−3^
Δρ_min_ = −0.28 e Å^−3^
Absolute structure: Flack (1983 ▶), 1345 Friedel pairs
Flack parameter: 0.01 (9)

### 

Data collection: *APEX2* (Bruker, 2007 ▶); cell refinement: *SAINT* (Bruker, 2007 ▶); data reduction: *SAINT*; program(s) used to solve structure: *SHELXS97* (Sheldrick, 2008 ▶); program(s) used to refine structure: *SHELXL97* (Sheldrick, 2008 ▶); molecular graphics: *ORTEP-3 for Windows* (Farrugia, 1997 ▶) and *Mercury* (Macrae *et al.*, 2006 ▶); software used to prepare material for publication: *publCIF* (Westrip, 2010 ▶).

## Supplementary Material

Crystal structure: contains datablocks I, global. DOI: 10.1107/S1600536810038274/zq2060sup1.cif

Structure factors: contains datablocks I. DOI: 10.1107/S1600536810038274/zq2060Isup2.hkl

Additional supplementary materials:  crystallographic information; 3D view; checkCIF report

## Figures and Tables

**Table 1:** Hydrogen-bond geometry (Å, °)

*D*—H⋯*A*	*D*—H	H⋯*A*	*D*⋯*A*	*D*—H⋯*A*
N4—H4*A*⋯O3^i^	0.85 (3)	2.21 (3)	2.993 (3)	153 (3)
N4—H4*A*⋯O4	0.85 (3)	2.39 (3)	2.924 (3)	121 (3)
N2—H2*A*⋯O4^ii^	0.88 (3)	2.03 (3)	2.848 (3)	155 (3)
C2—H2⋯O2^iii^	0.93	2.48	3.399 (4)	168
C13—H13⋯O3^i^	0.93	2.49	3.258 (3)	140

Symmetry codes: (i) 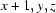; (ii) 
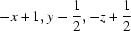
; (iii) 
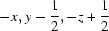
.

**Fig. 1.**
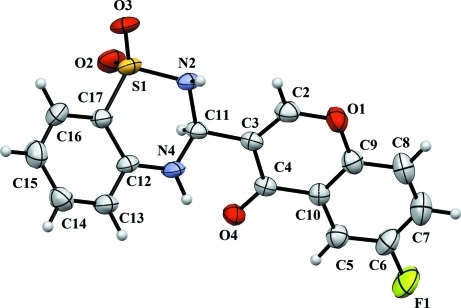
The molecular structure of the title molecule, with the atom-numbering scheme. Displacement ellipsoids are drawn at the 50% probability level.

**Fig. 2.**
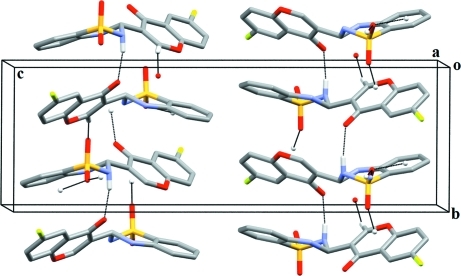
A packing diagram of the title compound showing hydrogen bonds as dashed lines.

## supplementary crystallographic information

### Comment

The 1,2,4-benzothiadiazine-1,1-dioxide ring system has attained considerable importance in pharmaceutical and medicinal chemistry mainly due to the compounds such as chlorothiazide and diazoxide (Zhu *et al.*, 2005; Kamal *et al.*, 2007*a*). The sulfonamide group is an active pharmacophore which is responsible for many biological activities (Weisman & Brown, 1964). The crystal structure of the condensation product of 4-aminobenzenesulfonamide with 4-oxo-4*H*-1-benzopyran-3-carboxaldehyde has previously been reported (al-Rashida *et al.*, 2009). Herein, we report the crystal structure of the condensation product of 2-aminobenzenesulfonamide with 6-fluoro-4-oxo-4*H*-1-benzopyran-3-carboxaldehyde.

In the molecule of the title compound (Fig. 1), the two rings of the chromone system (F1, O1, O4, C2—C10) are coplanar, making a dihedral angle of 0.55 (19)°. The carbon atom C11 deviates only by 0.034 (5) Å from the mean plane of the chromone. The phenyl ring (C12—C17) and the atoms N4, S1 and C11 are coplanar as well (rms deviation = 0.033) and make a dihedral angle of 54.28 (5)° with the mean plane of the chromone system.

The crystal structure is stabilized by intra- and intermolecular N—H···O and C—H···O hydrogen bonds which link the molecules into an infinite two-dimensional network (Fig. 2).

### Experimental

A solution of 2-aminobenzenesulfonamide (1.0 mmol) in 10 ml ethanol was slowly added to the stirred solution of 6-fluoro-4-oxo-4*H*-1-benzopyran-3-carboxaldehyde (1.0 mmol) containing a catalytic amount of *p*-toluene sulfonic acid (*p*-TsOH) and refluxed for 3 hrs. The resulting product was isolated by filtration, washed with ethanol, dried and recrystallized from hot ethanol and acetone (1:1) (yield 81%, m.p. 472 K).

### Refinement

The H atoms attached to N were located in a difference Fourier map and their coordinates were refined, with *U*_iso_(H) = 1.2*U*_eq_(N). The remaining H atoms were positioned geometrically with C-H = 0.93 and 0.98 Å for aromatic and methine H atoms, respectively, and constrained to ride on their parent atoms, with *U*_iso_(H) = 1.2*U*_eq_(C).

### Crystal data

**Table:**

C_16_H_11_FN_2_O_4_S	*F*(000) = 712
*M**_r_* = 346.34	*D*_x_ = 1.567 Mg m^−^^3^
Orthorhombic, *P*2_1_2_1_2_1_	Mo *K*α radiation, λ = 0.71073 Å
Hall symbol: P 2ac 2ab	Cell parameters from 1626 reflections
*a* = 7.0739 (3) Å	θ = 3.3–22.0°
*b* = 8.2861 (4) Å	µ = 0.26 mm^−^^1^
*c* = 25.0456 (12) Å	*T* = 296 K
*V* = 1468.05 (12) Å^3^	Needle, orange
*Z* = 4	0.31 × 0.06 × 0.05 mm

### Data collection

**Table:**

Bruker APEXII CCD area-detector diffractometer	1993 reflections with *I* > 2σ(*I*)
Radiation source: fine-focus sealed tube	*R*_int_ = 0.056
graphite	θ_max_ = 28.3°, θ_min_ = 3.0°
phi and ω scans	*h* = −9→9
9538 measured reflections	*k* = −10→10
3453 independent reflections	*l* = −33→24

### Refinement

**Table:**

Refinement on *F*^2^	Secondary atom site location: difference Fourier map
Least-squares matrix: full	Hydrogen site location: inferred from neighbouring sites
*R*[*F*^2^ > 2σ(*F*^2^)] = 0.052	H atoms treated by a mixture of independent and constrained refinement
*wR*(*F*^2^) = 0.093	*w* = 1/[σ^2^(*F*_o_^2^) + (0.0318*P*)^2^] where *P* = (*F*_o_^2^ + 2*F*_c_^2^)/3
*S* = 0.97	(Δ/σ)_max_ < 0.001
3453 reflections	Δρ_max_ = 0.23 e Å^−^^3^
223 parameters	Δρ_min_ = −0.28 e Å^−^^3^
3 restraints	Absolute structure: Flack (1983), 1345 Friedel pairs
Primary atom site location: structure-invariant direct methods	Flack parameter: 0.01 (9)

### Special details

**Table:**

Geometry. All esds (except the esd in the dihedral angle between two l.s. planes) are estimated using the full covariance matrix. The cell esds are taken into account individually in the estimation of esds in distances, angles and torsion angles; correlations between esds in cell parameters are only used when they are defined by crystal symmetry. An approximate (isotropic) treatment of cell esds is used for estimating esds involving l.s. planes.
Refinement. Refinement of *F*^2^ against ALL reflections. The weighted *R*-factor *wR* and goodness of fit *S* are based on *F*^2^, conventional *R*-factors *R* are based on *F*, with *F* set to zero for negative *F*^2^. The threshold expression of *F*^2^ > σ(*F*^2^) is used only for calculating *R*-factors(gt) *etc*. and is not relevant to the choice of reflections for refinement. *R*-factors based on *F*^2^ are statistically about twice as large as those based on *F*, and *R*- factors based on ALL data will be even larger.

### Fractional atomic coordinates and isotropic or equivalent isotropic displacement parameters (Å^2^)

**Table:**

	*x*	*y*	*z*	*U*_iso_*/*U*_eq_	
S1	0.05991 (10)	0.79823 (11)	0.18441 (3)	0.0371 (2)	
O2	0.0424 (3)	0.9692 (3)	0.18575 (10)	0.0520 (6)	
O3	−0.1066 (2)	0.7017 (3)	0.18043 (9)	0.0481 (6)	
N4	0.4758 (3)	0.7352 (4)	0.19622 (11)	0.0431 (8)	
H4A	0.593 (4)	0.736 (4)	0.2026 (12)	0.052*	
N2	0.1714 (3)	0.7397 (3)	0.23806 (11)	0.0315 (7)	
H2A	0.167 (4)	0.634 (4)	0.2387 (11)	0.038*	
C17	0.2141 (4)	0.7419 (4)	0.13334 (13)	0.0342 (8)	
C16	0.1469 (4)	0.7134 (5)	0.08251 (14)	0.0469 (9)	
H16	0.0194	0.7295	0.0753	0.056*	
C15	0.2643 (5)	0.6619 (4)	0.04245 (14)	0.0551 (10)	
H15	0.2187	0.6446	0.0081	0.066*	
C14	0.4523 (5)	0.6364 (5)	0.05458 (13)	0.0499 (10)	
H14	0.5335	0.6002	0.0279	0.060*	
C13	0.5222 (4)	0.6627 (4)	0.10455 (13)	0.0453 (10)	
H13	0.6496	0.6440	0.1113	0.054*	
C12	0.4050 (4)	0.7178 (4)	0.14604 (12)	0.0338 (8)	
C11	0.3657 (4)	0.8018 (4)	0.24047 (12)	0.0334 (7)	
H11	0.3629	0.9197	0.2374	0.040*	
C3	0.4590 (4)	0.7558 (4)	0.29238 (12)	0.0323 (8)	
C4	0.6462 (4)	0.8218 (4)	0.30203 (12)	0.0297 (7)	
O4	0.7261 (3)	0.9080 (3)	0.26942 (8)	0.0382 (6)	
C10	0.7285 (4)	0.7785 (4)	0.35354 (12)	0.0314 (7)	
C5	0.9080 (4)	0.8345 (4)	0.36782 (12)	0.0396 (9)	
H5	0.9778	0.8998	0.3449	0.048*	
C6	0.9767 (5)	0.7904 (5)	0.41614 (15)	0.0516 (11)	
F1	1.1503 (3)	0.8486 (3)	0.43125 (8)	0.0783 (8)	
C7	0.8798 (5)	0.6941 (5)	0.45116 (15)	0.0598 (11)	
H7	0.9314	0.6690	0.4843	0.072*	
C8	0.7072 (5)	0.6355 (4)	0.43699 (13)	0.0539 (10)	
H8	0.6406	0.5670	0.4596	0.065*	
C9	0.6331 (4)	0.6803 (4)	0.38801 (13)	0.0401 (8)	
O1	0.4555 (3)	0.6203 (3)	0.37625 (9)	0.0471 (6)	
C2	0.3784 (4)	0.6620 (4)	0.32923 (13)	0.0429 (9)	
H2	0.2584	0.6221	0.3217	0.051*	

### Atomic displacement parameters (Å^2^)

**Table:**

	*U*^11^	*U*^22^	*U*^33^	*U*^12^	*U*^13^	*U*^23^
S1	0.0215 (3)	0.0401 (5)	0.0498 (5)	0.0054 (4)	0.0011 (4)	0.0013 (5)
O2	0.0419 (12)	0.0430 (15)	0.0711 (17)	0.0132 (11)	0.0049 (13)	0.0072 (15)
O3	0.0186 (10)	0.0601 (16)	0.0658 (16)	−0.0026 (10)	−0.0003 (10)	−0.0045 (14)
N4	0.0170 (12)	0.075 (2)	0.0377 (18)	−0.0037 (14)	0.0000 (11)	−0.0042 (15)
N2	0.0221 (12)	0.0295 (17)	0.0430 (17)	−0.0023 (11)	0.0036 (11)	0.0033 (13)
C17	0.0262 (15)	0.038 (2)	0.039 (2)	0.0004 (14)	0.0012 (13)	0.0021 (16)
C16	0.0346 (16)	0.060 (3)	0.046 (2)	0.0012 (18)	−0.0116 (16)	0.007 (2)
C15	0.056 (2)	0.074 (3)	0.035 (2)	−0.007 (2)	−0.0062 (19)	0.000 (2)
C14	0.049 (2)	0.063 (3)	0.038 (2)	−0.001 (2)	0.0073 (18)	−0.005 (2)
C13	0.0280 (17)	0.063 (3)	0.044 (2)	−0.0004 (16)	0.0028 (15)	−0.002 (2)
C12	0.0215 (15)	0.041 (2)	0.039 (2)	−0.0034 (14)	−0.0012 (13)	−0.0002 (18)
C11	0.0238 (14)	0.038 (2)	0.0387 (19)	−0.0060 (15)	0.0045 (13)	0.0012 (17)
C3	0.0282 (14)	0.035 (2)	0.0341 (19)	−0.0021 (15)	0.0062 (14)	−0.0030 (16)
C4	0.0302 (15)	0.0255 (19)	0.0332 (19)	−0.0013 (14)	0.0044 (13)	−0.0031 (16)
O4	0.0351 (11)	0.0418 (15)	0.0376 (13)	−0.0113 (11)	0.0007 (10)	0.0056 (11)
C10	0.0374 (16)	0.026 (2)	0.0307 (18)	0.0027 (16)	0.0000 (14)	−0.0034 (16)
C5	0.0404 (19)	0.039 (2)	0.039 (2)	−0.0019 (15)	−0.0028 (15)	−0.0014 (17)
C6	0.046 (2)	0.060 (3)	0.048 (2)	0.000 (2)	−0.0181 (18)	−0.004 (2)
F1	0.0598 (13)	0.108 (2)	0.0671 (16)	−0.0153 (13)	−0.0323 (11)	0.0078 (14)
C7	0.077 (3)	0.062 (3)	0.041 (2)	−0.001 (2)	−0.016 (2)	0.006 (2)
C8	0.075 (3)	0.051 (3)	0.036 (2)	−0.003 (2)	0.0002 (19)	0.0075 (19)
C9	0.0470 (18)	0.036 (2)	0.038 (2)	−0.0005 (17)	0.0016 (17)	0.0016 (19)
O1	0.0512 (13)	0.0521 (16)	0.0381 (14)	−0.0150 (13)	−0.0022 (11)	0.0119 (12)
C2	0.0349 (16)	0.048 (3)	0.045 (2)	−0.0079 (16)	0.0031 (16)	−0.0028 (19)

### Geometric parameters (Å, °)

**Table:**

S1—O2	1.422 (2)	C11—C3	1.507 (4)
S1—O3	1.427 (2)	C11—H11	0.9800
S1—N2	1.632 (3)	C3—C2	1.335 (4)
S1—C17	1.745 (3)	C3—C4	1.453 (4)
N4—C12	1.360 (4)	C4—O4	1.224 (3)
N4—C11	1.463 (4)	C4—C10	1.460 (4)
N4—H4A	0.85 (3)	C10—C9	1.364 (4)
N2—C11	1.469 (3)	C10—C5	1.398 (4)
N2—H2A	0.88 (3)	C5—C6	1.354 (4)
C17—C16	1.379 (4)	C5—H5	0.9300
C17—C12	1.402 (4)	C6—C7	1.370 (5)
C16—C15	1.370 (5)	C6—F1	1.373 (3)
C16—H16	0.9300	C7—C8	1.361 (5)
C15—C14	1.380 (4)	C7—H7	0.9300
C15—H15	0.9300	C8—C9	1.385 (4)
C14—C13	1.363 (4)	C8—H8	0.9300
C14—H14	0.9300	C9—O1	1.383 (4)
C13—C12	1.406 (4)	O1—C2	1.343 (3)
C13—H13	0.9300	C2—H2	0.9300
			
O2—S1—O3	119.19 (14)	N2—C11—C3	110.9 (2)
O2—S1—N2	108.60 (15)	N4—C11—H11	109.1
O3—S1—N2	106.84 (13)	N2—C11—H11	109.1
O2—S1—C17	109.76 (14)	C3—C11—H11	109.1
O3—S1—C17	108.36 (14)	C2—C3—C4	119.6 (3)
N2—S1—C17	102.83 (13)	C2—C3—C11	123.8 (3)
C12—N4—C11	122.9 (2)	C4—C3—C11	116.6 (3)
C12—N4—H4A	122 (2)	O4—C4—C3	122.0 (3)
C11—N4—H4A	112 (2)	O4—C4—C10	123.3 (3)
C11—N2—S1	112.4 (2)	C3—C4—C10	114.7 (3)
C11—N2—H2A	112.3 (19)	C9—C10—C5	119.0 (3)
S1—N2—H2A	107.2 (19)	C9—C10—C4	120.5 (3)
C16—C17—C12	121.1 (3)	C5—C10—C4	120.4 (3)
C16—C17—S1	120.5 (2)	C6—C5—C10	117.7 (3)
C12—C17—S1	118.3 (2)	C6—C5—H5	121.1
C15—C16—C17	121.4 (3)	C10—C5—H5	121.1
C15—C16—H16	119.3	C5—C6—C7	123.3 (3)
C17—C16—H16	119.3	C5—C6—F1	118.2 (4)
C16—C15—C14	118.0 (3)	C7—C6—F1	118.4 (3)
C16—C15—H15	121.0	C8—C7—C6	119.3 (3)
C14—C15—H15	121.0	C8—C7—H7	120.3
C13—C14—C15	121.8 (3)	C6—C7—H7	120.3
C13—C14—H14	119.1	C7—C8—C9	118.3 (3)
C15—C14—H14	119.1	C7—C8—H8	120.8
C14—C13—C12	121.1 (3)	C9—C8—H8	120.8
C14—C13—H13	119.4	C10—C9—O1	121.9 (3)
C12—C13—H13	119.4	C10—C9—C8	122.2 (3)
N4—C12—C17	123.3 (3)	O1—C9—C8	115.8 (3)
N4—C12—C13	120.0 (2)	C2—O1—C9	117.6 (3)
C17—C12—C13	116.5 (3)	C3—C2—O1	125.6 (3)
N4—C11—N2	109.6 (3)	C3—C2—H2	117.2
N4—C11—C3	109.0 (2)	O1—C2—H2	117.2

### Hydrogen-bond geometry (Å, °)

**Table:**

*D*—H···*A*	*D*—H	H···*A*	*D*···*A*	*D*—H···*A*
N4—H4A···O3^i^	0.85 (3)	2.21 (3)	2.993 (3)	153 (3)
N4—H4A···O4	0.85 (3)	2.39 (3)	2.924 (3)	121 (3)
N2—H2A···O4^ii^	0.88 (3)	2.03 (3)	2.848 (3)	155 (3)
C2—H2···O2^iii^	0.93	2.48	3.399 (4)	168
C13—H13···O3^i^	0.93	2.49	3.258 (3)	140

Symmetry codes: (i) *x*+1, *y*, *z*; (ii) −*x*+1, *y*−1/2, −*z*+1/2; (iii) −*x*, *y*−1/2, −*z*+1/2.
